# Bioactive surface modifications on dental implants: a systematic review and meta-analysis of osseointegration and longevity

**DOI:** 10.1007/s00784-024-05958-y

**Published:** 2024-10-11

**Authors:** Maher AL Shayeb, Sittana Elfadil, Huda Abutayyem, Abedalrahman Shqaidef, Maria Maddalena Marrapodi, Marco Cicciù, Giuseppe Minervini

**Affiliations:** 1 Department of Clinical Sciences, College of Dentistry, Center of Medical and Bio-Allied Health Sciences Research, Ajman University, Ajman, UAE; 2 Department of Paediatric Dentistry and Orthodontics, Faculty of Dentistry, University of Jordan, Amman, Jordan; 3 Department of Woman, Child and General and Specialist Surgery, University of Campania “Luigi Vanvitelli”, Naples, 80121 Italy; 4 Department of Biomedical and Surgical and Biomedical Sciences, Catania University, Catania, 95123 Italy; 5 Multidisciplinary Department of Medical-Surgical and Odontostomatological Specialties, University of Campania “Luigi Vanvitelli”, Naples, 80121 Italy; 6 Saveetha Dental College and Hospitals, Saveetha Institute of Medical and Technical Sciences (SIMATS), Saveetha University, Chennai, Tamil Nadu India

**Keywords:** Bioactive surface modifications, Dental implants, Osseointegration, Implant longevity, Collagen coatings

## Abstract

**Background:**

Bioactive surface modifications have been proposed to enhance osseointegration and longevity of dental implants. This study aimed to systematically review and perform a meta-analysis on the effectiveness of various bioactive coatings in promoting bone integration and improving implant longevity.

**Methods:**

A systematic review was conducted, including studies that investigated bioactive surface modifications on titanium dental implants. Outcomes of interest were bone-to-implant contact (BIC) and implant longevity over a 30-day period. Data were extracted and analyzed using RevMan 5 (version 5.4.1), with forest plots generated to represent the mean difference (MD) and 95% confidence intervals (CI) under a random effects model.

**Results:**

The meta-analysis showed a significant improvement in BIC for surface-modified implants, with an overall MD of 7.29 (95% CI [2.94, 11.65]). Heterogeneity analysis indicated moderate heterogeneity (Tau² = 18.57, Chi² = 16.08, df = 8, *P* = 0.04, I² = 50%). The test for overall effect yielded Z = 3.28 (*P* = 0.001). For implant longevity, the overall MD was 7.52 (95% CI [3.18, 11.85]), with moderate heterogeneity (Tau² = 17.28, Chi² = 14.95, df = 8, *P* = 0.06, I² = 47%). The test for overall effect yielded Z = 3.40 (*P* = 0.0007).

**Conclusion:**

Bioactive surface changes significantly improved osseointegration and lifespan of dental implants. Collagen-based coatings consistently encouraged early bone integration, while BMP-2 combinations were effective for osseointegration. Optimizing bioactive agent doses and combinations was critical for achieving desired outcomes.

## Introduction

Dental implants have become a widely accepted and preferred solution for the replacement of missing teeth, offering functional and aesthetic benefits [1]. However, the success and longevity of these implants are highly dependent on effective osseointegration – the direct structural and functional connection between living bone and the surface of a load-bearing implant. Achieving optimal osseointegration is critical for implant stability and the long-term success of dental prosthetics [2]. To enhance this process, various surface modifications of dental implants have been developed, aiming to improve the interaction between the implant surface and the surrounding biological environment [3].

Surface modifications can be broadly categorized into physical, chemical, and biochemical alterations. Physical modifications include changes in surface topography and roughness, which have been shown to influence cell behavior and improve mechanical interlocking between the implant and bone [4, 5]. Chemical modifications involve the alteration of surface chemistry to enhance biocompatibility and promote bone cell attachment and proliferation. Biochemical modifications typically include the incorporation of bioactive molecules, such as growth factors, peptides, and proteins, which can further enhance bone regeneration and integration [6–8].

In the context of rodent studies, which are commonly used in dental implant research, “implant longevity” has generally been as survival beyond 30 days of age in mice, which is a significant milestone in their lifespan. This definition is based on the fact that mice have a relatively short median lifespan of around 18–24 months, and 30 days represents a substantial portion of their early life [9, 10].

The concept of bioactivity in surface modifications refers to the ability of the implant surface to elicit a specific biological response at the interface, leading to enhanced bone formation and integration [8]. Bioactive surfaces are designed to mimic the natural extracellular matrix, providing cues that promote cellular adhesion, proliferation, and differentiation. This bioactivity is often achieved through the application of coatings, such as hydroxyapatite, bioactive glass, or other osteoconductive and osteoinductive materials, which have been extensively studied for their potential to improve osseointegration in terms of bone-to-implant contact (BIC) [5, 11].

Despite the advancements in surface modification techniques, there remains considerable variability in the reported outcomes of these modifications concerning osseointegration and implant longevity. The heterogeneity in study designs, implant types, surface treatment methods, and evaluation criteria complicates the ability to draw definitive conclusions about the most effective surface modifications. Therefore, this systematic review and meta-analysis aimed to critically assess the evidence on the effectiveness of bioactive surface modifications of dental implants concerning osseointegration and longevity.

## Materials and methods

### Eligibility criteria

The PRISMA guidelines [12] were followed to ensure the transparency and rigor of this systematic review and meta-analysis. Two independent reviewers conducted the screening and selection process to minimize bias and ensure consistency. Discrepancies between reviewers were resolved through discussion, and if necessary, a third reviewer was consulted.

The PECO (Population, Exposure, Comparator, Outcome) protocol for this review was clearly defined to guide the selection of studies. The population (P) comprised animals who received dental implants for tooth replacement. The exposure (E) was defined as dental implants with bioactive surface modifications, including but not limited to coatings with hydroxyapatite, bioactive glass, growth factors, and other osseoconductive or osseoinductive materials. The comparator (C) group included dental implants without bioactive surface modifications, such as those with standard surfaces or alternative non-bioactive modifications. The primary outcome (O) of interest was osseointegration, measured by parameters such as bone-implant contact (BIC), bone volume density (BVD), and other histomorphometric analyses.

The inclusion and exclusion criteria for this review devised for this review are as follows:

*Inclusion criteria*:

1. **Study design**: Only in-vivo studies were included to ensure clinical relevance and applicability.
2. **Population**: Studies involving animal subjects who received dental implants for tooth replacement were considered.
3. **Intervention**: Studies that investigated dental implants with bioactive surface modifications, such as hydroxyapatite coatings, bioactive glass, growth factors, peptides, and other osseoconductive or osseoinductive materials, were included.
4. **Comparators**: Studies that compared bioactive surface-modified implants with implants having standard surfaces or alternative non-bioactive modifications were selected.
5. **Outcomes**: Studies that reported on osseointegration outcomes, including BIC, BVD, and other histomorphometric analyses, as well as implant longevity outcomes, such as survival rates and stability over time, were included.
6. **Publication status**: Peer-reviewed articles published in English were considered to ensure the quality and accessibility of the studies.

*Exclusion criteria*:

1. **Study design**: Case reports, reviews, and editorials were excluded to maintain the focus on clinically relevant human data.
2. **Population**: Studies involving patients with systemic conditions or diseases that could significantly affect bone metabolism and implant integration, such as uncontrolled diabetes or osteoporosis, were excluded.
3. **Intervention**: Studies that did not specifically investigate bioactive surface modifications or that involved experimental modifications not widely recognized in the scientific community were excluded.
4. **Outcomes**: Studies that did not provide specific data on osseointegration or implant longevity, or that reported only subjective outcomes without quantitative measures, were excluded.
5. **Publication status**: Non-peer-reviewed articles, conference abstracts, theses, and dissertations were excluded to ensure the inclusion of rigorously vetted research.

### Database search strategy

The search strategy was executed across six major electronic databases: PubMed, Scopus, Web of Science, Embase, Cochrane Library, and Google Scholar. Boolean operators and Medical Subject Headings (MeSH) keywords were systematically employed to capture all pertinent literature as elucidated through Table 1.

**Table 1:** Search phrases and keywords utilised across the different databases

Database	Search string
PubMed	(“Dental Implants“[MeSH] OR “Implant Dentistry“[MeSH] OR “Tooth Implants“[MeSH]) AND (“Surface Properties“[MeSH] OR “Surface Modification“[MeSH] OR “Surface Coating“[MeSH]) AND (“Osseointegration“[MeSH] OR “Bone-Implant Interface“[MeSH] OR “Biocompatible Coated Materials“[MeSH] OR “Bone Regeneration“[MeSH]) AND (“Longevity“[MeSH] OR “Survival Rate“[MeSH] OR “Long-Term Efficacy“[MeSH])
Scopus	(TITLE-ABS-KEY(“dental implants” OR “implant dentistry” OR “tooth implants”) AND TITLE-ABS-KEY(“surface properties” OR “surface modification” OR “surface coating”) AND TITLE-ABS-KEY(“osseointegration” OR “bone-implant interface” OR “bone regeneration”) AND TITLE-ABS-KEY(“longevity” OR “survival rate” OR “long-term efficacy”))
Web of Science	(TS=(“dental implants” OR “implant dentistry” OR “tooth implants”) AND TS=(“surface properties” OR “surface modification” OR “surface coating”) AND TS=(“osseointegration” OR “bone-implant interface” OR “biocompatible coated materials” OR “bone regeneration”) AND TS=(“longevity” OR “survival rate” OR “long-term efficacy”))
Embase	(‘dental implant’/exp OR ‘implant dentistry’/exp OR ‘tooth implant’/exp) AND (‘surface property’/exp OR ‘surface modification’/exp OR ‘surface coating’/exp) AND (‘osseointegration’/exp OR ‘bone implant interface’/exp OR ‘bone regeneration’/exp OR ‘biocompatible coating material’/exp) AND (‘longevity’/exp OR ‘survival rate’/exp OR ‘long term efficacy’/exp)
Cochrane Library	((“dental implants” OR “implant dentistry” OR “tooth implants”) AND (“surface properties” OR “surface modification” OR “surface coating”) AND (“osseointegration” OR “bone-implant interface” OR “bone regeneration” OR “biocompatible coated materials”) AND (“longevity” OR “survival rate” OR “long-term efficacy”)):ti, ab, kw
Google Scholar	(allintitle: “dental implants” OR “implant dentistry” OR “tooth implants”) AND (allintitle: “surface properties” OR “surface modification” OR “surface coating”) AND (allintitle: “osseointegration” OR “bone-implant interface” OR “bone regeneration” OR “biocompatible coated materials”) AND (allintitle: “longevity” OR “survival rate” OR “long-term efficacy”)

### Data extraction protocol

The data extraction protocol for this review involved the use of a standardized data extraction form, which was developed prior to the commencement of data extraction to maintain consistency and reduce bias. Two independent reviewers were assigned to perform the data extraction to ensure reliability and to cross-verify the extracted information. Any discrepancies between the reviewers were resolved through discussion, and if consensus could not be reached, a third reviewer was consulted.

### Bias assessment protocol

The bias assessment protocol was designed using the Systematic Review Centre for Laboratory animal Experimentation (SYRCLE) Risk of Bias tool [13]. Although this tool was originally developed for animal studies, its structured approach to assessing methodological quality and potential biases was adapted for in-vivo human studies in this review.

### Statistical analysis protocol

The meta-analysis protocol was designed and executed using Review Manager (RevMan) version 5.4.1. The primary aim was to quantitatively synthesize the data on the impact of bioactive surface modifications on dental implants, specifically focusing on osseointegration and longevity outcomes. The meta-analysis was conducted under the assumption of a random-effects (RE) model, which is appropriate given the expected heterogeneity among the included studies. The analysis included the generation of forest plots to represent the mean differences (MD) with 95% confidence intervals (CI).

## Results

### PRISMA study selection process

**Fig. 1:**
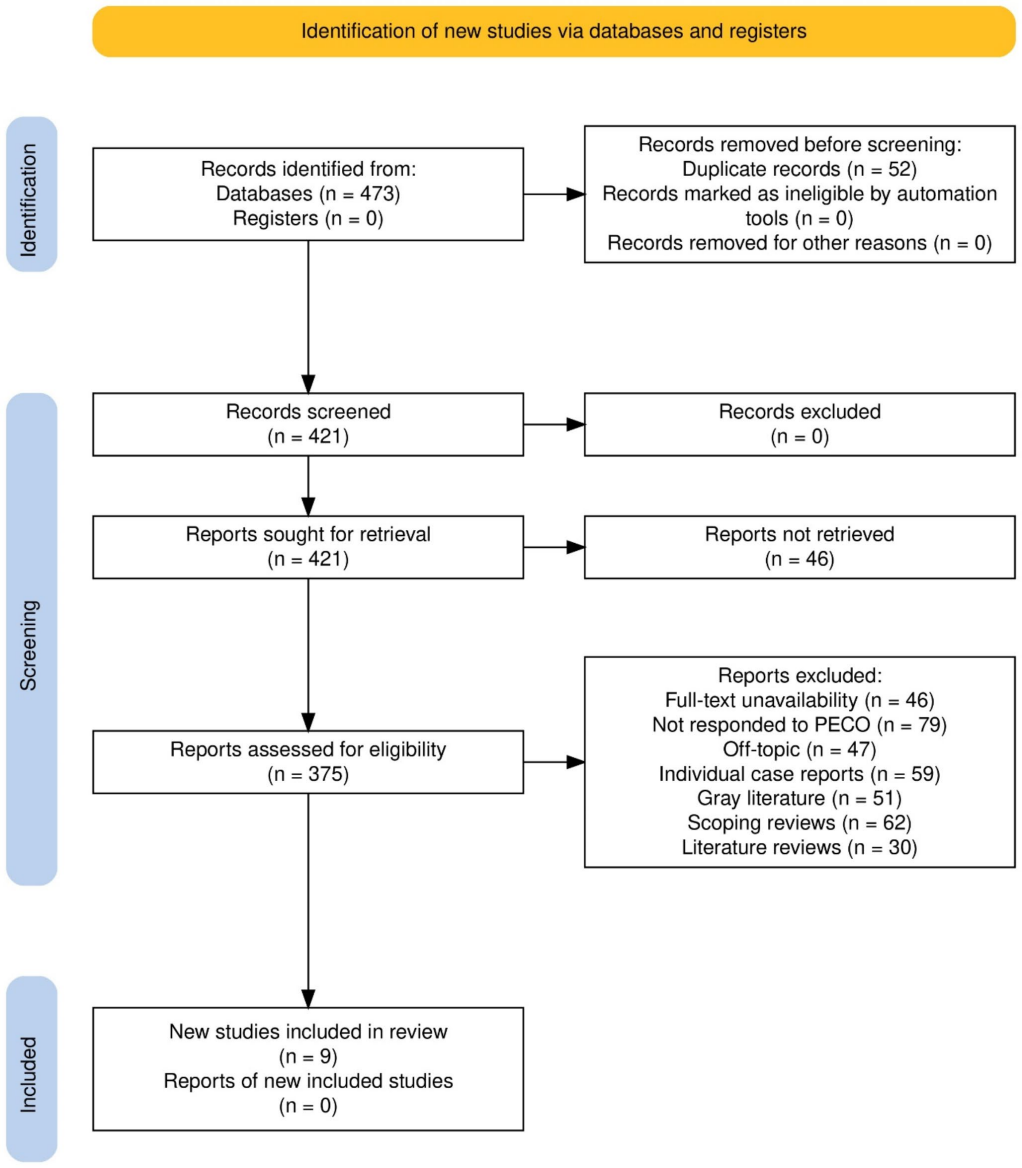
Study selection process for the review

The study selection process for this systematic review was thorough and methodical, adhering to established protocols to ensure the inclusion of relevant and high-quality studies. Initially, 473 records were identified through database searches, with no additional records found in registers. Prior to screening, 52 duplicate records were removed, leaving 421 records for initial screening. No records were marked as ineligible by automation tools or removed for other reasons at this stage. During the screening phase, all 421 records were evaluated based on their titles and abstracts. This process resulted in no exclusions at this stage, and all 421 records were sought for full-text retrieval. However, 46 reports could not be retrieved, reducing the number of reports available for full-text assessment to 375. The full-text assessment phase involved a detailed evaluation of these 375 reports against the predefined inclusion and exclusion criteria. This phase led to the exclusion of several studies for various reasons: 46 reports were excluded due to full-text unavailability despite attempts to obtain them; 79 reports did not meet the PECO criteria; 47 reports were off-topic; 59 were individual case reports; 51 were grey literature; 62 were scoping reviews; and 30 were literature reviews. Ultimately, 9 studies [14–22] met al.l the inclusion criteria and were included in the final review. (Fig. 1)

### Assessed bias observations

**Fig. 2:**
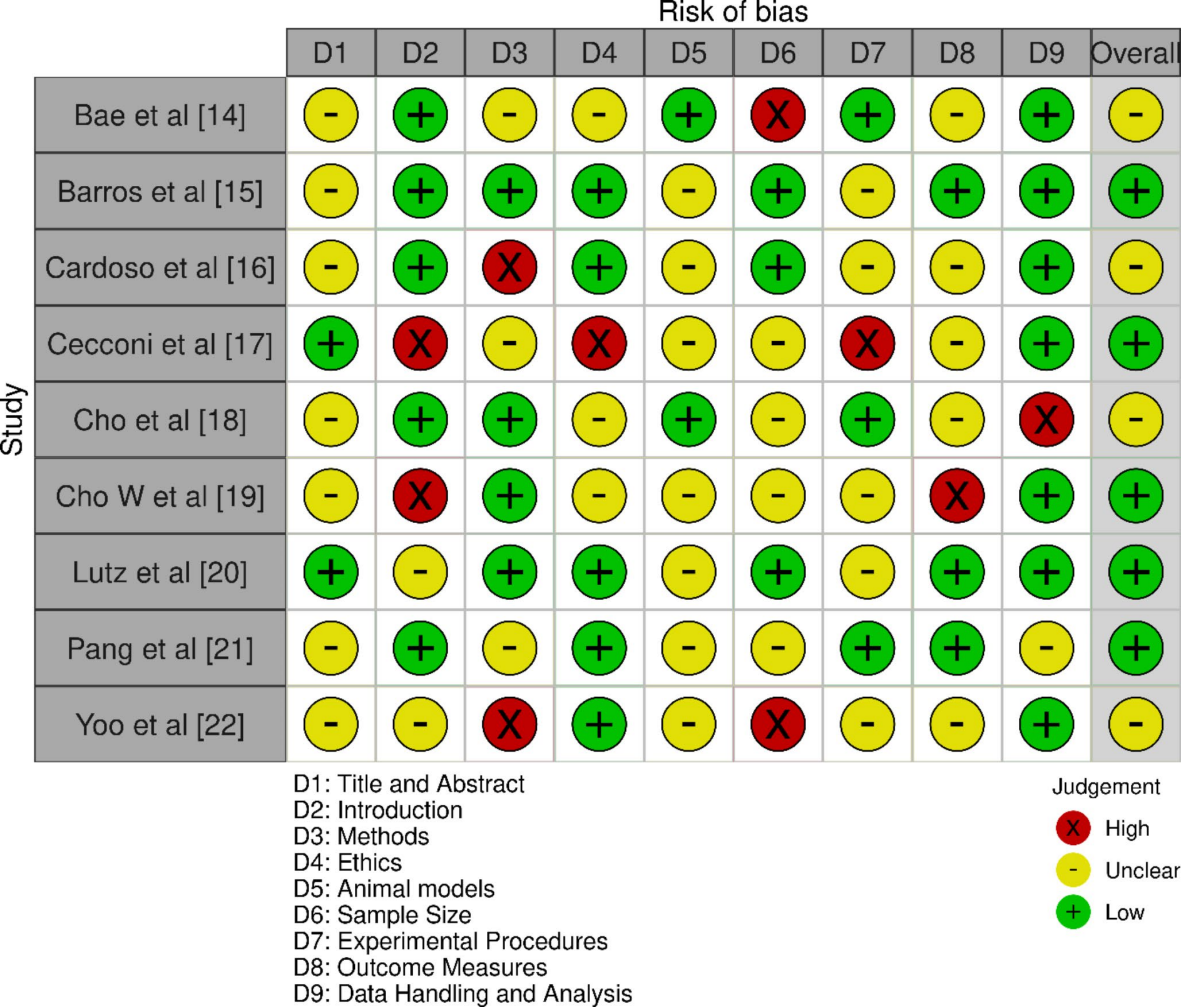
Bias assessment done across the selected studies

The bias assessment across the selected studies revealed varying levels of bias in different categories (Fig. 2). Bae et al. [14] and Cardoso et al. [16] exhibited moderate overall bias, with specific concerns in methods and ethics. Barros et al. [15], Lutz et al. [20], and Pang et al. [21] demonstrated low overall bias, showing more consistency across categories. Cecconi et al. [17], despite having low overall bias, had high bias in introduction and ethics. Cho et al. [18] and Yoo et al. [22] had moderate overall bias, with some high bias noted in sample size and methods respectively. Cho W et al. [19] showed low overall bias, though high bias was observed in introduction and outcome measures.

### Selected studies and their baseline characteristics

The studies included in Table 2 elucidate the different surface modification agents on different sample types and implantation locations over varied study durations across the included studies [14–22]. Bae et al. [14] investigated the use of Type I Collagen/GA on rat tibiae over an extended period of 84 weeks. This long-term study aimed to evaluate the sustained impact of the collagen coating on bone integration and implant stability.

Barros et al. [15] utilized bioactive peptides on dog mandibles with an 8-week study duration. This research focused on how bioactive peptides could enhance osseointegration and bone healing in a relatively short period. Cardoso et al. [16] examined PPL10BMP in pig parietal bones over 4 and 8 weeks. This study aimed to determine the efficacy of PPL10BMP in promoting bone regeneration and implant stability in a time-dependent manner.

Cecconi et al. [17] explored Type I Collagen/Apatite on rabbit femurs over 7 weeks. The combination of collagen and apatite was assessed for its potential to improve bone formation and attachment to the implant surface. Cho et al. [18] evaluated vitronectin-derived peptides on rabbit tibiae for 2 weeks. This short-term study investigated the initial effects of the peptide on bone tissue scaffolding and early-stage osseointegration.

Cho W et al. [19] studied Type I Collagen/GA on dog jaws over an 8-week period. This research aimed to compare the efficacy of gamma-irradiated collagen crosslinking with glutaraldehyde crosslinking in enhancing bone regeneration around implants. Lutz et al. [20] focused on biomimetic active peptide (P-15) in the forehead region of pigs over 2 and 4 weeks. This study sought to understand the early effects of P-15 on bone contact and density around the implant site.

Pang et al. [21] assessed the combination of BMP-2 and HA on rabbit tibiae over 4 weeks. The study aimed to determine the synergistic effects of BMP-2 and hydroxyapatite in promoting osseointegration and bone healing. Yoo et al. [22] investigated rhBMP-2/PLGA on rabbit tibiae over 3 and 7 weeks. This study focused on the initial and intermediate effects of the combination on bone integration and implant stability.

**Table 2:** Demographic characteristics of the included papers

Study ID	Year	Surface modification agent	Sample type utilised	Implantation location	Study duration
Bae et al. [14]	2018	Type I Collagen/GA	Rat	Tibia	84 weeks
Barros et al. [15]	2009	Bioactive peptide	Dog	Mandible	8 weeks
Cardoso et al. [16]	2017	PPL10BMP	Pig	Parietal bone	4, 8 weeks
Cecconi et al. [17]	2014	Type I Collagen/Apatite	Rabbit	Femur	7 weeks
Cho et al. [18]	2019	Vitronectin-derived peptide	Rabbit	Tibiae	2 weeks
Cho W et al. [19]	2021	Type I Collagen/GA	Dog	Jaw	8 weeks
Lutz et al. [20]	2010	Biomimetic active peptide (P-15)	Pig	Forehead region	2 and 4 weeks
Pang et al. [21]	2021	BMP-2 + HA	Rabbit	Tibiae	4 weeks
Yoo et al. [22]	2015	rhBMP-2/PLGA	Rabbit	Tibiae	3 and 7 weeks

### Implant parameters assessed

Table 3 shows the specific details of the devices used, the material and quantity of implants, and the measured parameters across the included papers [14–22]. Bae et al. [14] used devices measuring 2.5 × 1.5 mm made of titanium (Ti) with a sample size of 12. The study focused on BIC and new bone volume (NBV). The findings indicated that the surface modification promoted substantial bone integration and increased new bone volume around the implants.

Barros et al. [15] employed implants sized 9.5 × 4.5 mm composed of pure titanium (48). The measured parameters were BIC and bone density (BD). The results demonstrated enhanced bone-to-implant contact and improved bone density, suggesting effective osseointegration and bone formation. Cardoso et al. [16] utilized devices of 6 × 1.1 mm made of pure titanium, with a total of 120 implants. The study measured bone-to-tissue (B/T) and BIC. The findings showed a significant increase in both parameters, indicating effective integration of the implant with the surrounding bone tissue.

Cecconi et al. [17] used implants measuring 8.5 × 4 mm made of titanium (24). The primary parameter measured was BIC. The study reported improved bone-to-implant contact, indicating a beneficial effect of the surface modification on early bone integration. Cho et al. [18] investigated implants of 11 × 3.5 mm made of grade 4 titanium (16). The parameters measured included BIC and bone area (BA). The results highlighted enhanced BIC and increased bone area, suggesting effective early-stage osseointegration and bone growth.

Cho W et al. [19] utilized larger implants, 8 × 40 mm, made of pure titanium (36). The study focused on BIC and BA. The findings indicated a significant improvement in both parameters, demonstrating effective bone integration and growth around the implants. Lutz et al. [20] used devices measuring 8 × 3.5 mm made of pure titanium (54). The parameters measured were BIC and BD. The results showed enhanced bone-to-implant contact and increased bone density, supporting the effectiveness of the surface modification in promoting bone integration.

Pang et al. [21] employed implants sized 7 × 3.3 mm composed of pure titanium (8). The study measured BIC, BA, and removal torque (RTQ). The findings demonstrated improvements in all parameters, indicating effective osseointegration, bone growth, and implant stability. Yoo et al. [22] used implants measuring 7 × 3.75 mm made of pure grade IV titanium (32). The measured parameters included BIC and BA. The study reported significant improvements in both parameters, highlighting the positive impact of the surface modification on bone integration and growth.

**Table 3:** Technical characteristics of the modification agent and its observed impact on implants

Study ID	Device specifics	Material & quantity	Measured parameters	Summary of findings
Bae et al. [14]	2.5 × 1.5 mm	Ti (12)	BIC, NBV	Radiation cross-linked collagen-coated Ti implants showed osteoinductive qualities without adverse effects of chemical agents.
Barros et al. [15]	9.5 × 4.5 mm	Pure Ti (48)	BIC, BD	Bone apposition and density around Ti implants varied with bioactive peptide concentrations.
Cardoso et al. [16]	6 × 1.1 mm	Pure Ti (120)	B/T, BIC	PPL10 and BMP-2 combination did not enhance bone formation.
Cecconi et al. [17]	8.5 × 4 mm	Ti (24)	BIC	Coating with bone apatite and type I collagen increased new bone formation and attachment around Ti implants.
Cho et al. [18]	11 × 3.5 mm	Ti, grade 4 (16)	BIC, BA	Tissue scaffolding at 2 weeks, increased bone density at 4 weeks. No significant differences in BIC and BA between groups.
Cho W et al. [19]	8 × 40 mm	Pure Ti (36)	BIC, BA	Gamma-irradiated collagen crosslinking was as effective as GA crosslinking for bone regeneration.
Lutz et al. [20]	8 × 3.5 mm	Pure Ti (54)	BIC, BD	Positive impact on BIC with high contact rates at 14 and 30 days. No significant effect on peri-implant BD.
Pang et al. [21]	7 × 3.3 mm	Pure Ti (8)	BIC, BA, RTQ	BMP-2 combined with HAP activated osseointegration.
Yoo et al. [22]	7 × 3.75 mm	Pure grade IV Ti (32)	BIC, BA	PLGA/rhBMP-2 Ti coatings increased BIC during early healing.

### Osseointegration outcomes assessed

**Fig. 3:**
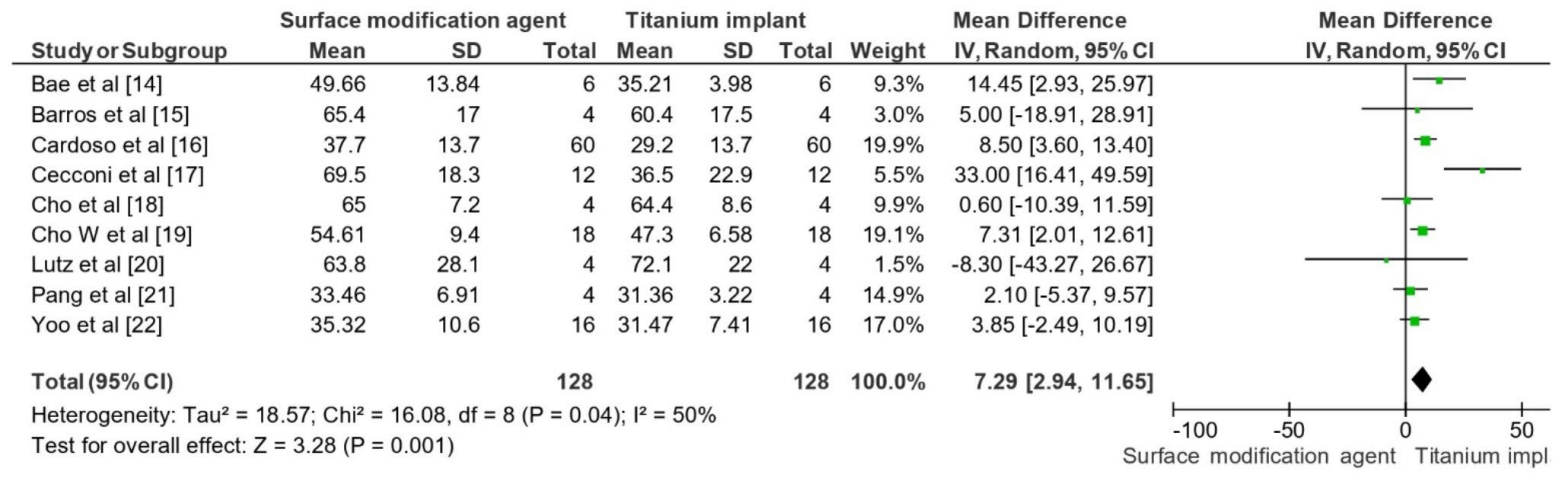
Impact of surface modification agent on dental implant in terms of osseointegration (BIC) across a 30-day period

The meta-analysis revealed a statistically significant improvement in BIC for surface-modified implants compared to unmodified titanium implants (Fig. 3). The overall mean difference (MD) was 7.29 with a 95% CI of [2.94, 11.65], indicating that surface modifications positively influenced osseointegration. The heterogeneity analysis showed a Tau² value of 18.57, a Chi² value of 16.08 with 8 degrees of freedom (*P* = 0.04), and an I² value of 50%, suggesting moderate heterogeneity among the studies. The test for overall effect yielded a Z value of 3.28 (*P* = 0.001), confirming the significant impact of bioactive surface modifications on enhancing BIC. These findings demonstrated the efficacy of surface modifications in improving the osseointegration of dental implants.

### Longevity outcomes assessed

**Fig. 4:**
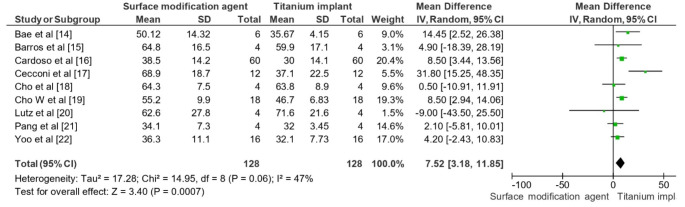
Impact of surface modification agent on dental implant in terms of longevity across a 30-day period

The meta-analysis revealed a statistically significant improvement in the longevity of dental implants with bioactive surface modifications compared to unmodified titanium implants over a 30-day period (Fig. 4). The overall MD was 7.52 with a 95% CI of [3.18, 11.85]. This indicated that surface-modified implants performed better in terms of longevity. The heterogeneity analysis showed a Tau² value of 17.28, a Chi² value of 14.95 with 8 degrees of freedom (*P* = 0.06), and an I² value of 47%, suggesting moderate heterogeneity among the studies. The test for overall effect yielded a Z value of 3.40 (*P* = 0.0007), confirming the significant positive impact of bioactive surface modifications on the longevity of dental implants within the studied period.

## Discussion

Nanostructured coatings such as calcium, calcium phosphate, and HA have been extensively utilized on implants. These coatings can be applied to metal implants through methods like hydrothermal deposition or plasma spraying. These materials release calcium and phosphate ions, which promote mineralization of the interface tissues and facilitate bone healing [23]. Additionally, inorganic coatings influence stress transmission to the bone during masticatory functions, ensuring proper force distribution during repeated cycles [23].

BMPs have been shown to play a crucial role in regulating and promoting osteogenic and bone mesenchymal stem cells, leading to their increased application in dental coatings [24]. The use of recombinant human BMPs (rhBMPs) has been approved by the FDA for therapeutic use in dentistry, with rhBMP2 being commercially available and utilized for bone regeneration in dental implantology [24]. In our review, we explored the use of bioactive surface modifications on dental implants, including BMPs, to enhance implant osseointegration and bone formation. Notably, our findings suggest that BMP-2 combined with HAP can activate osseointegration, as demonstrated by Pang et al. [21]. This is consistent with previous studies that have shown the effectiveness of BMP-2 in promoting bone formation and regeneration [25].

However, our study also highlights the challenges associated with protein form delivery, including dosage challenges and the need for repeated applications [25]. In contrast, gene delivery of rhBMP2 has been shown to facilitate protein synthesis for extended periods, as demonstrated by Yoo et al. [22]. This approach may offer a more promising solution for promoting bone formation and regeneration. When compared to our study, the use of rhBMP2 in dental coatings has been shown to promote excellent osseointegration, as demonstrated by [26, 27]. However, our study suggests that alternative bioactive surface modifications, such as collagen-based coatings, may offer similar or improved outcomes, as demonstrated by Bae et al. [14] and Cho W et al. [19].

Another approach to enhance biocompatibility on implant surfaces involves the accumulation of ECM proteins. During the bone integration growth phase, fibroblast growth factor stimulates fibroblasts to secrete various ECM proteins, such as elastin, chondroitin sulfate, collagen, fibronectin, hyaluronic acid, and other proteoglycans [24]. For example, one study [28] showed that collagen-chondroitin sulfate coatings significantly increased osseointegration by promoting bone formation at the implant–bone interface. Another paper [29] tested mussel adhesive protein, which enhanced osseointegration by promoting the differentiation of bone-forming cells and improving cell adhesion and proliferation. Raphael et al. [30] demonstrated that elastin-like protein coatings on implants in rat tibia and femur reduced micromovements associated with deficient force loads, thereby improving mechanical properties through rapid osseointegration. Additionally, it has also been found that incorporating hyaluronic acid into polyelectrolyte multilayer coatings enhanced osteogenic differentiation of adipose-derived stem cells and increased bone mineral deposition [31, 32].

When comparing these findings to those of Lopez-Valverde et al. [33], both studies emphasized the positive impact of surface modifications on osseointegration, particularly during the early stages of healing. They found BMPs to be the most favorable coating, which aligns with our findings regarding the effectiveness of BMP-2 combinations. However, they noted very high heterogeneity (I² = 99%), indicating more variability in their pooled studies compared to our moderate heterogeneity levels. Meng et al. [34] reviewed biologically active dental implant surfaces and reported that biomolecular coatings, including BMPs, improved peri-implant bone formation and osseointegration during early healing stages. This is consistent with our findings, particularly regarding the positive effects of BMP-2. Both studies called for long-term clinical validation, acknowledging that results from animal studies may not directly translate to human clinical outcomes.

Kligman et al. [35] discussed various implant surface modifications aimed at enhancing osseointegration and reducing biofilm formation. While our study focused on specific bioactive coatings, Kligman et al. [35] provided a broader overview of physical, chemical, and biological techniques. Both studies underscored the importance of modifying implant surfaces to improve clinical outcomes, though Kligman et al. [35] highlighted a wider range of materials and methods beyond our scope. Han et al. [36] summarized the effects of different surface modification methods on osseointegration and biofilm attachment. Their review covered techniques such as plasma spraying and anodic oxidation, which were not the focus of our study. However, both studies shared the goal of improving implant success rates by enhancing osseointegration and minimizing complications such as biofilm formation. They also discussed the mechanical, chemical, and biological disadvantages of various methods, providing a more comprehensive evaluation of surface modification techniques.

### Limitations

This study had several limitations that need to be considered when interpreting the findings. Firstly, the heterogeneity among the included studies was moderate, as indicated by the I² values of 50% for bone-to-implant contact (BIC) and 47% for implant longevity. This heterogeneity suggests variability in study designs, surface modification techniques, and animal models, which may have influenced the outcomes. Secondly, the sample sizes in some studies were relatively small, potentially affecting the robustness and generalizability of the results.

Additionally, the follow-up period for assessing implant longevity was limited to 30 days, which may not fully capture the long-term effects of bioactive surface modifications on dental implants. The variations in measurement techniques and reporting standards for BIC and other parameters across studies also posed challenges in standardizing the data for meta-analysis. Moreover, the review included a range of bioactive agents and coating methods, and while this diversity provides a broad overview, it also complicates direct comparisons and specific conclusions about the efficacy of individual modifications.

### Future implications and relevance

While our review was based on animal studies, the findings suggest that bioactive surface modifications may be worth exploring further in human studies. The results of our review highlight the need for additional research into the optimal design and application of bioactive surface modifications for dental implants. Future studies can build upon our findings to investigate the safety and efficacy of these modifications in human clinical trials.

## Conclusion

Our findings demonstrate that bioactive surface modifications on dental implants significantly improve osseointegration and implant longevity in animal models. Notably, collagen-based coatings consistently promoted early bone integration, while combinations involving BMP-2 were effective in enhancing osseointegration. However, the benefits of bioactive surface modifications varied depending on the specific bioactive agent and coating method used, highlighting the importance of optimizing concentrations and combinations of bioactive agents for achieving optimal outcomes. Despite some heterogeneity among the included studies, the positive impact of bioactive surface modifications on dental implant performance was evident. These findings have important implications for the development of more effective dental implants and underscore the need for further research to translate these findings to human clinical trials.

## Data Availability

No datasets were generated or analysed during the current study.
